# Red blood cell transfusion is a predictor of mortality and morbidity in children undergoing cardiac surgery

**DOI:** 10.1186/cc12303

**Published:** 2013-03-19

**Authors:** C Colognesi, R Maia, L Hajjar, F Bergamin, J Fukushima, E Osawa, J Almeida, L Camara, S Zeferino, V Guimaraes, F Galas

**Affiliations:** 1 Heart Institute, São Paulo, Brazil

## Introduction

Red blood cell (RBC) transfusion is associated with morbidity and mortality in critically ill patients. Congenital cardiac surgeries are associated with high rates of bleeding and consequently with high rates of allogeneic transfusion. We aimed to evaluate the association of transfusion with worse outcomes in children undergoing cardiac surgery.

## Methods

We performed a prospective cohort study of 205 patients undergoing cardiac surgery for congenital heart disease. We recorded baseline characteristics, RACHS-1 score, intraoperative data, transfusion requirement and severe postoperative complications as need for reoperation, acute kidney injury, arrhythmia, severe sepsis, septic shock, bleeding, stroke, and death during 30 days. We performed univariate analysis using baseline, intraoperative and postoperative variables. Selected variables (*P <*0.10) were included in a forward stepwise multiple logistic regression model to identify predictive factors of a combined endpoint including 30-day mortality and severe complications.

## Results

One hundred and thirty-six patients (66.3%) were exposed to RBC transfusion. In the intraoperative room, 63.4% of patients received at least one RBC unit, and in the ICU, 11.2% of children were transfused. From all patients, 66 (32.1%) presented the combined endpoint. Patients with complications had higher RACHS-1 score, were younger (69 months (0 to 137) vs. 73 months (37 to 138), *P <*0.001), had a lower weight (13 kg (3 to 23) vs. 20 kg (12 to 36), *P <*0.001), a longer time of surgery (475 minutes (410 to 540) vs. 353 (275 to 433), *P <*0.001), a longer duration of cardiopulmonary bypass (205 minutes (175 to 235) vs. 106 minutes (73 to 123), *P *= 0.003), a lower SVO_2 _at the end of surgery (59% (IQR 39 to 80) vs. 78% (71 to 83), *P <*0.001), a higher arterial lactate at the end of surgery (6.9 mmol/l (4.3 to 9.2) vs. 2.7 mmol/l (73 to 123), *P *= 0.003), a lower intraoperative hematocrit (26.2 ± 5.6% vs. 29.5 ± 6% (*P <*0.001)) and a lower hematocrit at the end of surgery (33.4 ± 6.7% vs. 36.9 ± 6.9% (*P <*0.001)) as compared with patients without complications. Patients with complications were more exposed to RBC transfusion in the intraoperative room (75% vs. 57%, *P *= 0.011) and in the ICU (21% vs. 6.4%, *P *= 0.002). In an adjusted model of logistic regression, RBC transfusion is an independent risk factor of combined endpoint (OR 4.25 (95% CI, 1.359 to 13.328), *P *= 0.013).

## Conclusion

Blood transfusion after pediatric cardiac surgery is a risk factor for worse outcome including 30-day mortality. Avoiding blood transfusion must be a goal of best postoperative care.

